# Characteristics of Haptic Peripersonal Spatial Representation of Object Relations

**DOI:** 10.1371/journal.pone.0160095

**Published:** 2016-07-27

**Authors:** Ryo Wako, Saho Ayabe-Kanamura

**Affiliations:** 1 Graduate School of Comprehensive Human Sciences, University of Tsukuba, Tsukuba, Ibaraki, Japan; 2 School of Integrative and Global Majors, Empowerment Informatics Program, University of Tsukuba, Tsukuba, Ibaraki, Japan; 3 Division of Psychology, University of Tsukuba, Tsukuba, Ibaraki, Japan; Centre de Neuroscience Cognitive, FRANCE

## Abstract

Haptic perception of space is known to show characteristics that are different to actual space. The current study extends on this line of research, investigating whether systematic deviations are also observed in the formation of haptic spatial representations of object-to-object relations. We conducted a haptic spatial reproduction task analogous to the parallelity task with spatial layouts. Three magnets were positioned to form corners of an isosceles triangle and the task of the participant was to reproduce the right angle corner. Weobserved systematic deviations in the reproduction of the right angle triangle. The systematic deviations were not observed when the task was conducted on the mid-sagittal plane. Furthermore, the magnitude of the deviation was decreased when non-informative vision was introduced. These results suggest that there is a deformation in spatial representation of object-to-object relations formed using haptics. However, as no systematic deviation was observed when the task was conducted on the mid-saggital plane, we suggest that the perception of object-to-object relations use a different egocentric reference frame to the perception of orientation.

## Introduction

The high ability to coordinate the hand with vision allows us to reach and grasp a coffee cup, catch a baseball with our hands, and defend ourselves from approaching harm through the use of our hands. Such coordination of the hand and vision are conducted so naturally that we do not consciously distinguish between visual and haptic spatial representations. However, there are numerous studies which show that spatial perception with vision and spatial perception with the hand (haptics) yield a different spatial representation. This can be observed from certain actions of daily life; for instance you may fail to accurately grasp a cup of coffee while keeping eye contact with a friend during a conversation. In such a situation the position of the coffee cup in visual coordinates must be recalculated into haptic coordinates, which seems to make the task a whole lot harder. In fact, bimodal neurons have been found which code visual stimuli in relation to the body and not in relation to the retina ([1,2]; on macaque monkeys; [3]; on humans), which again shows the importance of the hand being in sight for coordination of vision and haptics. However, there are situations where the hand cannot be attended to with sight (e.g. driving) and therefore understanding of the characteristics of haptic spatial representations are needed. The present study aims to investigate the formation of spatial representation of the surroundings (peripersonal space) through the use of haptics, in order to further understand the role of haptics in forming peripersonal spatial representations.

When considering the formation of haptic spatial representations, one must consider two spatial frames of reference: the egocentric reference frame and the allocentric reference frame. In the egocentric reference frame an object’s location is defined in relation to the observer’s body, whereas in the allocentric reference frame the object’s location is defined independent of the observer’s body [4]. In both reference frames an object’s location may be defined in terms of orientation and distance; the object’s orientation and distance from the observer for the egocentric reference frame, and the object’s orientation and distance from another object for the allocentric reference frame.

Through the parallelity task, deviations have been shown in the haptic perception of orientation. In the parallelity task participants are required to align two bars (one fixed reference bar and one freely moving test bar) to the felt parallel while being blindfolded. The parallelity task has revealed two characteristics of haptic spatial perception: the haptic oblique effect (see [5] for review) and the systematic deviations in the perception of parallelity [6–11]. Perception of horizontal or vertical orientation in Euclidean coordinates outperform oblique orientations (haptic oblique effect). The haptic oblique effect is observed not only on the horizontal plane but also on the mid-sagittal plane and the frontal planes [12]. Such superiority in perception of horizontal and vertical orientations as compared to oblique orientations suggest that haptic perception of space use Euclidean coordinate system to code orientations. However, along with the observation of the haptic oblique effect, systematic deviations which seem to reflect the use of a non-veridical egocentric reference frame are also observed from the parallelity task. When test bars are located to the right of a reference bar, clockwise rotations are perceived as parallel; anti-clockwise rotations are perceived as parallel when test bars are located to the left. The egocenter in the perception of orientation is shown to lie on the hand, as the posture of the hand rotates in a clockwise manner when perceiving bar orientations to the right [13,14]. To further support this view, no rotational deviations are observed when participants make two bars mirror symmetric using two hands as the posture of the left and right hands show mirror relations [6,15]. Investigations on three-dimensional haptic parallelity perception have also shown that the hand-centered egocentric reference frame best describe the systematic deviations [14]. These systematic deviations caused by the posture of the hand are not only observed on the horizontal plane but also on both the mid-sagittal and the frontoparallel planes [16,17]. It has also been found that accuracy in aligning bars to the subjective vertical is higher as compared to when aligning the rod to the gravitational vertical [18]. There is also a reference frame defined with coordinates in relation to the body as posture affects the perception in the vertical direction [19,20]. The posture of the hand (or use of the egocentric reference frame) highly affected the perception of orientation, resulting in systematic deviations, showing that haptic perception of spatial perception is not veridical.

Haptic perception of distance has been investigated by measuring performance in the perception of arm position. People tend to overestimate visually perceived reachable space [21] and proprioception is used to perceive the location of the arm in space [22], showing greater deviations when arm postures are at extreme postures such as full flexion or full contraction [23]. Localization of arm location is more precise in the radial direction with respect to the shoulder than in the azimuthal direction [24], as hand location is coded in a reference frame with the shoulder in use as the egocenter [25]. Perception of arm position become prone to errors when the arm is positioned more than 20cm from the body midline [26].

Scene recognition deals with perception of both orientation and distance, therefore it is an effective way to investigate haptic perception of object-to-object relations. Haptics is capable of perceiving and recognizing object-to-object relations as accurately as vision [27,28]. However, when the modality changes between learning and recognition, error rates increase showing that there is a cost when converting perceived space through vision or haptics to the other modality [27–29].

Previous researches on orientation and object location have highlighted the limitations in the haptic domain. The magnitude of the effects is in some cases very large, yet under everyday situations we are not aware of such deviations. This is because in most cases, spatial information concerning object in peripersonal space are perceived through interaction between vision and haptics. Altered visual feedback of arm position shifts perceived reachable space in the direction of the altered feedback and such changes in perceived reachable space were achieved in only a few trials [21]. Investigations into the perception of orientation have also shown that a weighted average of the egocentric and allocentric reference frames are used [8,30] and non-informative vision and delay between learning and reproduction decreases the magnitude of the deviation as both are known to promote heavier weightings to the allocentric reference frame [10,11,31]. Integration of data from visual only and haptic only trials can predict a participant’s performance in visual-haptic trials using a Bayesian model [32]. Taken together, it is evident that simultaneous acquisition of spatial information through haptics and vision is crucial for Euclidean spatial representation to be formed. However, under situations where visual information concerning hand location is not acquired (e.g., while driving a car), the characteristics caused by haptic original limitations are not calibrated and therefore deviations between spatial representations formed from haptics and vision remain.

The current study aims to investigate the characteristics of haptic spatial representation of several objects located in space. Perceiving the location of the hand and perceiving the locational relations of objects are similar yet very different. In both, a single object may be expressed in terms of a single spatial coordinate in relation to the body. However, when perceiving the relation of several object locations, one object's location may be expressed relative to other object’s locations (object-to-object relation), therefore objects may be expressed in different spatial coordinates depending on where the object is coded in relation to. This type of perception of object-to-object relation is allocentric in nature. Such allocentric coding of object location is crucial for trajectory planning of the arm as it is not always the case that the arm moves from the center of the body to the target object. Accurate planning of arm trajectories from one object to another require the use of one objects orientation in relation to another object as well as the distance between the two objects. From the results of previous research on orientation, it may be hypothesized that there is a deformation in the perception of object-to-object relations. However, since investigations concerning object-to-object relations have only been investigated through scene recognition, details concerning how object-to-object relations are deformed are not yet clear. Therefore, we conducted a task where participants were required to reproduce a learned object-to-object relation and examined whether any systematic deformations (or deviations) were observed (experiment 1). We confirmed the use of a different egocenter by introducing factors which affect movement of the arm (e.g. gravity) and observed its effects on the perception of object-to-object relations (experiment 2). Finally, any deviation of reproduced object positions should reflect the effects of the egocentric reference frame in haptic perception of space, therefore experimental manipulations which shift weightings to the allocentric reference frame should decrease the amount of deviation observed (experiment 3).

## Experiment 1

Experiment 1 aimed to investigate the characteristics of perception of object-to-object relations using haptics. Previous research has focused on the investigation of haptic spatial representation of object-to-object relations through recognition. The task of the current study was designed to observe reproduction of object-to-object relations in order to further understand the characteristics of haptic spatial representations.

### Methods

The experimental procedure was approved by the ethics board of the University of Tsukuba.

#### Participants

Twenty undergraduate and graduate students (ten for the right hand group and nine for the left hand group; mean age ± SD = 20.73±2.12) from University of Tsukuba volunteered to participate in this experiment. All participants were right handed. Written informed consent was obtained from all individual participants included in the study.

#### Experimental design

A three factor mixed experimental design with arrangement (oblique, horizontal-vertical) and reproduction space (front space, right space or left space) as within participant factors and hand (right, left) as between participant factor.

#### Stimuli and apparatus

**Fig 1:**
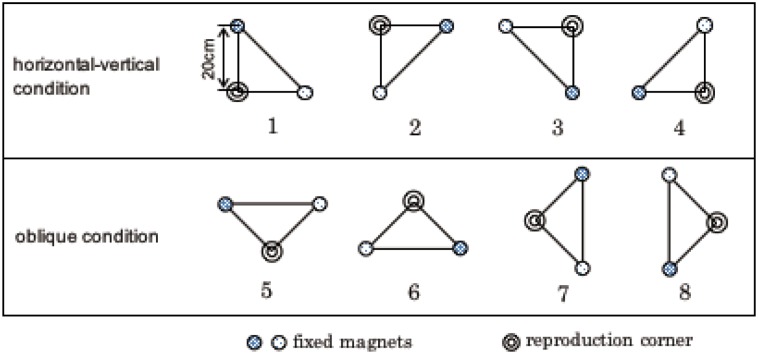
A schematic diagram of the 8 layouts used in the experiment. For each layout, the right angle corner was the corner which the participant reproduced.

**Fig 2:**
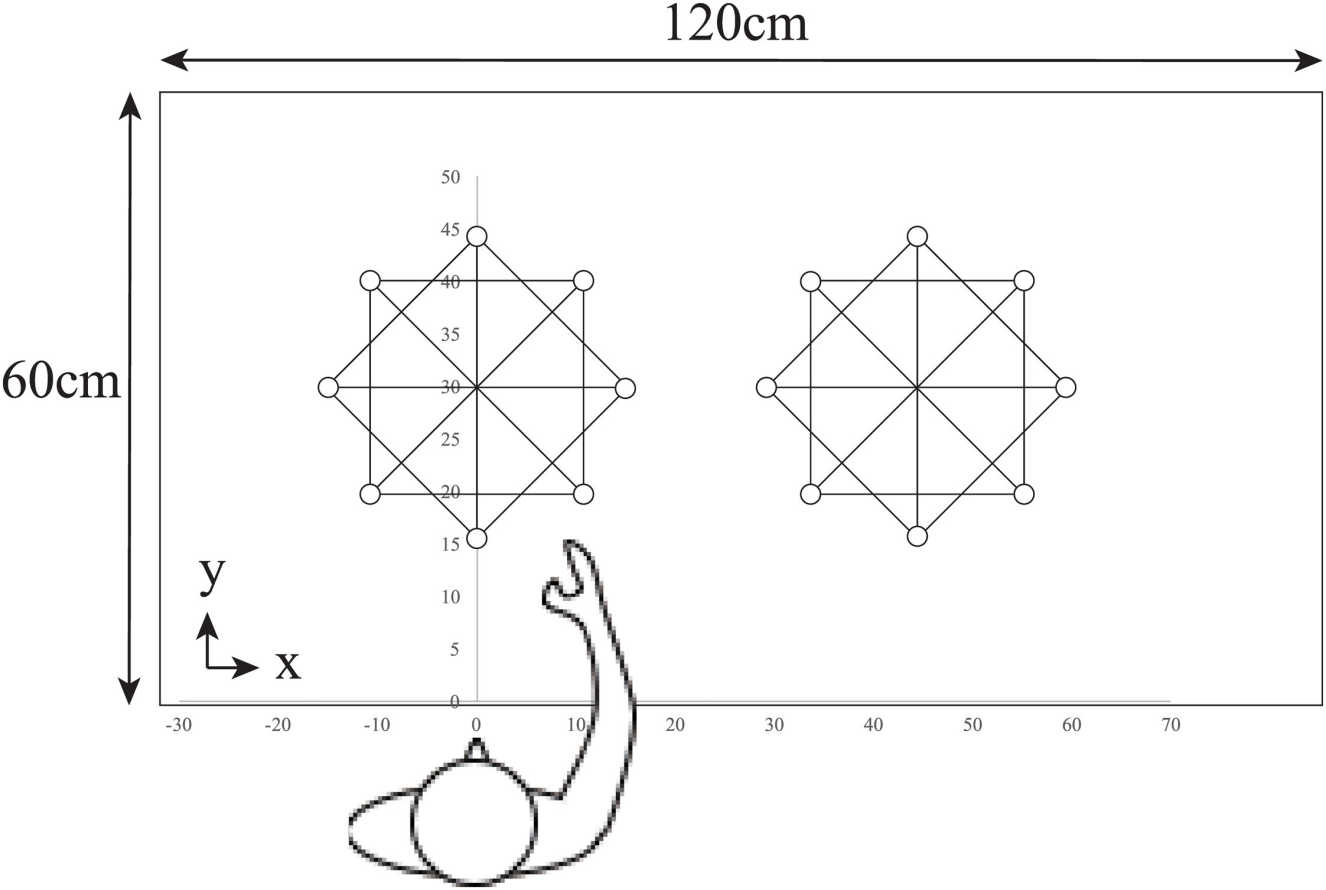
A schematic diagram of the experimental setup for the right handed group. The rectangle represents the table. The white circles depict possible places where magnets could be located. Three out of the eight positions were used to make an isosceles triangle. Participants wore eye masks throughout the experiment.

Stimuli were three magnets presented on a table to form an isosceles triangle. The three magnets were presented so as to form 8 different layouts (Fig 1). The magnets were 4cm in diameter, each with different textures (aluminum foil, cotton, beads). Different textures were used so that each texture matched each corner of the isosceles triangle so that the participants were able to know which corner they were touching. The two 45 degree corner magnets were placed 20cm away from the right angle corner magnet. A length of 20cm was chosen so that the participant could not touch two magnets at once with their hand and therefore needed to move their arm in order to perceive the relation between the three magnets. The 8 layouts were further split into two arrangement conditions: the oblique condition and the horizontal-vertical condition. Oblique and horizontal-vertical conditions were defined by the orientation of the right angle corner with respect to the other two corners. Therefore, in the oblique condition the right angle corner was placed 45 degrees to the other two corners and in the horizontal-vertical condition the right angle corner was placed 0 or 90 degrees to the other two corners. These conditions were chosen to investigate whether haptic perception of object relations are coded in terms of orientation and distance. If object positions are coded in terms of orientation, then accurate performance in the horizontal-vertical condition would be observed, similar to the haptic oblique effect. The layouts were positioned in the front space and right space as shown in Fig 2. The table was split into two, to resemble the two workspaces (front and right/left). Out of the eight white circles in Fig 2, three were used for each trial so as to form an isosceles triangle as shown in Fig 1. The three magnets were always presented in the front space during the learning phase and all three magnets were removed after learning had finished. Two magnets in the 45 degree corner were presented either in the front space (same position as in the learning phase) or in the right space (a shift of 45cm in the horizontal direction) during the reproduction phase, while the right angle corner magnet was handed to the participant to be reproduced. The front and right space were 45cm apart. Participants in the left hand group were seated in front of the right layouts depicted in Fig 2 and conducted the experiment with their left hand in the front space and left space.

#### Procedure

Participants sat in front of the experimental setup and were informed about the experimental procedure and gave written informed consent. After giving written informed consent, participants were instructed about the experimental procedure in detail.

The participants first sat in front of a table, either on the right side or the left depending on the group (Fig 2). They were instructed that the experiment consisted of a learning phase and a reproduction phase in which positional relations of three magnets were to be learned and reproduced while wearing an eye mask.

Participants next wore the eye mask and searched the tabletop for the three magnets with different textures on the side they were sitting (right of left). They had no time limit to search and learn the relation of the three magnets placed on the table. They used their right or left hand, depending on the group. They were asked to learn the spatial relation of the three magnets among each other, and not the position of the magnets in relation to the edges or sides of the table. Once the participants learned and were comfortable with the relation of the three magnets on the table, they placed their hand on their lap. At this stage, the experimenter told the participants to which side of the table the reproduction phase would take place. At the same time, the experimenter repositioned the 45 degree corner magnets either in front of them or on the right/left of the participant, depending on the condition. Participants were next handed one of the three magnets. Then they were required to position the handed magnet to complete the relation of the three magnets that they learned beforehand (two of the three magnets were already positioned and fixed on the instructed side of the table, either in front or on the right/left). The handed magnet was always the magnet that was positioned on the right angle corner of the isosceles triangle which participants previously learned. Furthermore, the textures and the relative positions of each magnet were matched between the learning and reproduction phases. This was done to make sure the participants were able to know which magnet position they were touching during the reproduction phase. Participants had no time limit to positioning the handed magnet to complete the relation of the three magnets and they were allowed to freely move the positioned magnet until they were comfortable with its position. Participants were free to move their hand back and forth between the three magnets to confirm its position. Participants moved their hand back to their lap once they were pleased with the position of the magnet. The above procedure was 1 trial and there were a total of 40 trials.

The experimenter recorded the position of the placed magnet by marking the center of the positioned magnet on the table surface. Only the displacement of the right angle corner magnet was taken into account because the other two magnets were already fixed to the table in the accurate positions. The other two magnets were fixed to the table to act as landmarks or cues in reproducing the right angle corner magnet. The other two magnet positions (45 degree angle) were not used to measure performance in the current task as the relative position of the 45 degree angle corner to the other two corners will not be the same. The 45 degree corner will be in the horizontal or vertical direction in relation to the right angle corner, however will be in the oblique direction in relation to the other 45 degree angle. This will mean that the layouts would not be able to be categorized into horizontal-vertical or oblique conditions. For these reasons the 45 degree angle corner was not measured. There were a total of 40 trials (8 arrangements, two repetitions in the front space and three repetitions in the right/left space) and took about one hour to complete. There were two repetitions in the front space as compared to three repetitions in the right (left) space due to time constraints. 1 hour was the maximum time that the participant was able to conduct the experiment without feeling stress while being blindfolded. It has been shown that brief glimpses of the hand [33] and non-informative vision [10] promote accurate perception of space while using haptics. This would mean that taking the blindfold off in between blocks of trials would change performance between trials after taking off the blindfold and trials before taking off the blindfold. For this reason, two trials were conducted in the front space while three were conducted in the right (left) space. The 40 trials were presented in random order. Participants in the right hand group used their right hand to learn and reproduce the layouts while participants in the left hand group used their left hand to learn and reproduce the layout.

### Results

**Fig 3:**
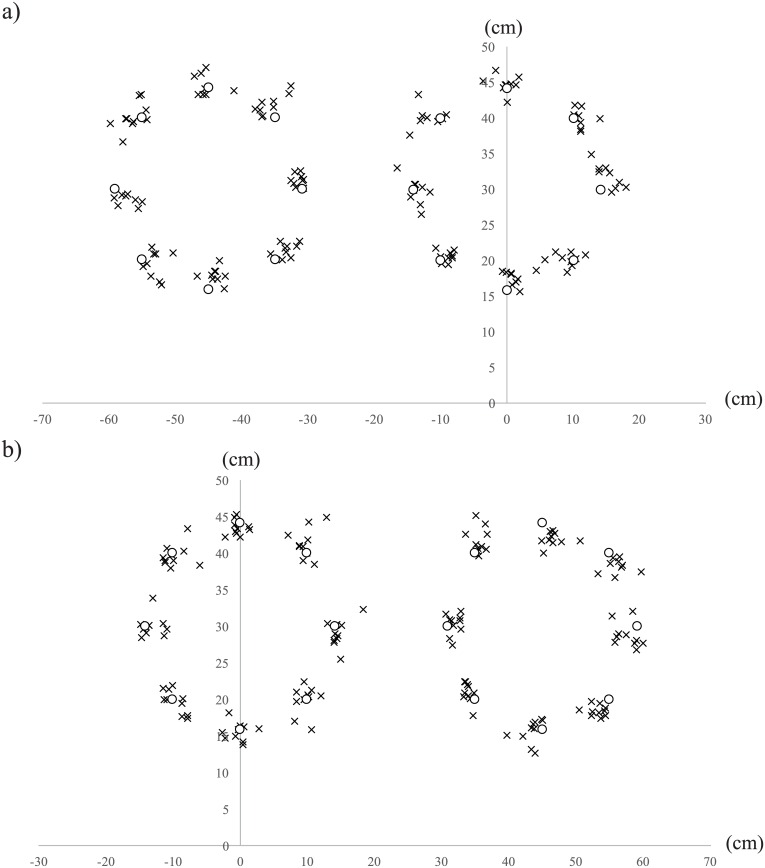
Scatter diagram of the mean positions of the reproduced magnet for each layout for each participant (crosses). White circles show the accurate positions of the magnets. 0 shows the position of the participant. a) Left hand group (*N* = 9) and b) Right hand group (*N* = 10).

A scatter diagram of the reproduced magnet position for the 8 layouts in the right, left, front spaces for each participant is shown in Fig 3. The points (one point for each layout) of each participant is the mean of the three replications for left and right spaces and the two replications for the front spaces.

**Fig 4:**
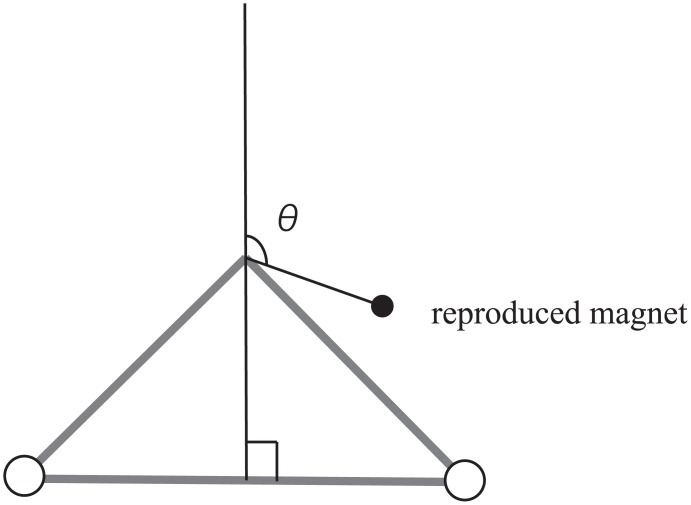
Schematic diagram of the measured angle of the reproduced magnet with respect to the accurate position. The top right area depicts angular deviations in the range of 0 to 90 degrees, bottom right area depicts angular deviations in the range of 90 to 180 degrees (a rightward rotational deviation), the bottom left area depicts angular deviations in the range of 180 to 270 degrees (a leftward rotational deviation), and the top left area depicts angular deviations in the range of 270 to 360 degrees.

Locations of the reproduced magnets are observed as deviations from the accurate position, measured in terms of x and y axes and angles. The x axis is the horizontal direction (positive values show rightward deviation with respect to the accurate position) and the y axis is the depth direction (positive values show deviations away from the accurate position), both with respect to the participant (Fig 2). Deviations in the x and y axes were used to investigate the magnitude of the deviation and direction. Angles were used to analyze the directional deviation. Fig 4 shows an example of the measured angle. One participant from the left hand group was excluded from the analysis as the data was more than 3SD away from the mean.

#### Magnitude of deviations in reproduction space

The absolute deviation shows the amount of deviation independent of the direction. A three factor (hand used, reproduction space, arrangement) mixed ANOVA was conducted on each axis. There was a significant main effect of reproduction space in both axes (x axis: *F*(1,17) = 11.93, *p* < .01; y axis: *F*(1,17) = 16.22, *p* < .001) with greater deviations in the right or left space than in the front space (First column Table 1). There were no main effects or interactions between the left, right hands used and arrangements.

**Table 1:** Absolute deviations of the reproduced magnet for all three experiments (cm).

			arrangement		arrangement	
			(oblique)		(horizontal-vertical)	
			x axis	y axis	x axis	y axis
Exp 1		reproduction space	1.82	2.1	2.14	1.64
	hand	(left)	-0.21	-0.31	-0.24	-0.13
	(left)	reproduction space	1.59	1.67	1.82	1.34
		(front)	-0.24	-0.14	-0.24	-0.13
		reproduction space	2.04	1.94	1.92	1.91
	hand	(right)	-0.18	-0.19	-0.27	-0.28
	(right)	reproduction space	1.36	1.43	1.29	1.57
		(front)	-0.18	-0.14	-0.19	-0.3
			arrangement		arrangement	
			(oblique)		(horizontal-vertical)	
			z axis	y axis	z axis	y axis
Exp 2		reproduction space	3.21	1.3	2.02	1.87
		(above shoulder)	-0.54	-0.22	-0.18	-0.2
		reproduction space	2.19	1.09	1.14	1.65
		(shoulder height)	-0.43	-0.2	-0.23	-0.33
			x axis	y axis		
Exp 3		visual cue	1.98	1.92		
	reproduction space	(blindfolded)	-0.22	-0.23		
	(right)	visual cue	1.43	1.46		
		(table hidden)	-0.17	-0.18		
		visual cue	1.33	1.5		
	reproduction space	(blindfolded)	-0.18	-0.23		
	(left)	visual cue	1.26	1.19		
		(table hidden)	-0.17	-0.14		

Numbers in brackets show *SE*

#### Direction of deviations

**Fig 5:**
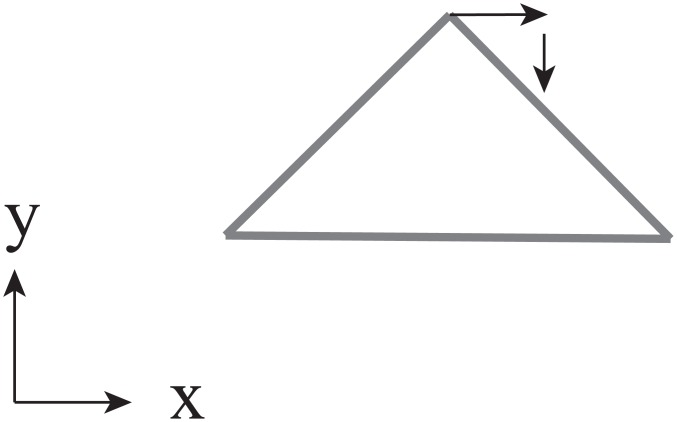
An example of the positive directions for each axis in each condition from a top view (rightward rotation).

**Fig 6:**
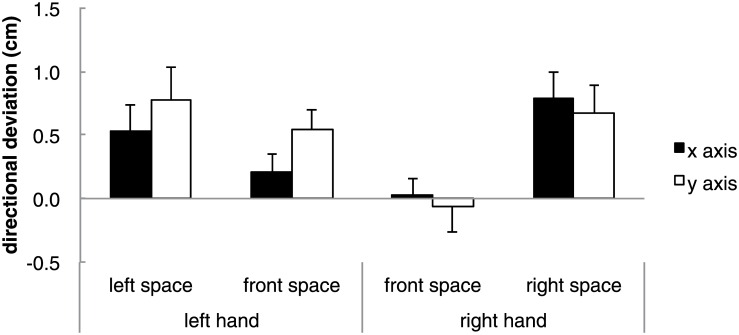
Mean directional deviations for each axis in each space for the left hand and right hand. Error bars show *SE*.

We next investigated whether the deviations showed any systematic character. Previous literature on haptic perception of orientation have shown that orientations perceived away from the body in the horizontal direction are perceived to be rotated (rightward rotations for orientations perceived in space to the right of the body and leftward rotations for orientations perceived to the left of the body). It may be hypothesized that the layouts in the present study may also be perceived as being rotated. First we calculated directional deviations. Calculated positive directional deviations in both axes show that the deviations show rightward rotational deviations for the right hand group and leftward rotational deviations for the left hand group. ANOVAs between the different groups and conditions were not conducted as the objective of computing directional deviation was not to examine in which axis or conditions deviation was the greatest, but was to consider whether the magnitude of the observed systematic deviations from the scatter diagram were statistically significant. The following directions were calculated as positive for each layout; layout 1 negative x axis and positive y axis, layout 2 positive x axis and positive y axis, layout 3 positive x axis and negative y axis, layout 4 negative x axis and negative y axis, layout 5 negative x axis and positive y axis, layout 6 positive x axis and negative y axis, layout 7 positive x axis and positive y axis, and layout 8 negative x axis and negative y axis. Fig 5 shows an example of the positive directions for a rightward rotation for a layout. A one-sample *t* test was carried out on the means of the directional deviation for each axis (x and y) in each reproduction space (front space, right space or left space). Significant positive directional deviations from zero were observed in both axes for right and left space (right space x axis: *t*(9) = 3.90, *p* < .01; right space y axis: *t*(9) = 3.15, *p* < .05; left space x axis: *t*(8) = 2.50, *p* < .05; left space y axis: *t*(8) = 3.07, *p* < .05). There was also a significant positive directional deviation in the front space for the left hand group in the y axis (*t*(8) = 3.51, *p* < .01) (Fig 6). Next, angular deviations were used to confirm that the reproduced positions of the magnets were shifted in the hypothesized rotational direction. The angles were divided into four areas representing each combination of x and y axis deviation (angles 0 to 90 degrees show positive x and y axis, angles 90 to 180 degrees show positive x and negative y axis, angles 180 to 270 degrees show negative x and y axis, and angles 270 to 360 degrees show negative x and positive y axis). Table 2 shows the frequency (8 layouts × 9 participants for left hand group and 10 participants for the right hand group) of magnets that were reproduced in each of the four areas. A chi-squared test of goodness-of-fit was performed to determine whether participants equally reproduced magnets in each area. Reproduction of the magnet was not equally distributed in the four areas for both the left (χ^2^ (3, *N* = 72) = 94.11, *p* < 0.01) and right spaces (χ^2^ (3, *N* = 80) = 35.83, *p* < 0.01) but not in the front space of both groups. Furthermore, except for layouts 5, 7 and 8 in the right space, all other layouts from the right and left spaces showed significant differences.

**Table 2:** The frequency of mean trials reproduced in the four areas.

		0~90°	90~180°	180~270°	270~360°
Hand	Reproduction space (left)	10	0	53	9
(left)	Reproduction space (front)	17	16	20	19
Hand	Reproduction space (right)	16	8	8	39
(right)	Reproduction space (front)	23	17	14	18

### Discussion

The aim of experiment 1 was to investigate the characteristics in perception and reproduction of object-to-object relations.

There were no effects of arrangement and therefore we did not observe any superiority in perception of vertical and horizontal relations as compared to oblique relations. This was different to the perception of orientation where observation of the haptic oblique effect is robust. However, our results did show greater deviation in the reproduced location of the magnet when reproduction was conducted away from the body (to the right or to the left), which is in line with earlier studies. These results were similar to the results investigating perception of orientation and perception of arm location, showing that haptic perception of space is prone to the locational relation of the object to the body. Greater deviations in unlearned spaces also match past literature on motor learning which have shown that motor learning is space specific and does not generalize to different locations in space [34,35]. We also observed deviations which we will discuss in terms of rotations. The directions of the deviations did not reflect the body-to-object relation (direction of deviation was not in the radial direction with respect to the shoulder). Rather, the deviations were significant in magnitude in the direction as if the whole layout was rotated in a certain direction (rightward for layouts in the right space and leftward for the left space), therefore showing a body-to-layout relation. The perceived orientation between the two fixed magnets might have been perceived as being rotated, similar to the perception of orientation [6,9–11]. A rotated perception of orientation between the two fixed magnets would lead to the third magnet to be reproduced in a rotational direction, resulting in the reproduced magnet to be placed in a position as if the whole layout had been rotated.

Participants in the current experiment were faced with the task of forming an object-to-object representation using feedback from arm movements, and then using this representation to calculate the desired motor commands to achieve the same trajectories as those experienced in the learning phase. In order for accurate calculation of motor commands to be achieved, accurate translation of arm location (in reference to the body and therefore egocentric) into Euclidean coordinates (allocentric) must be done. It has been shown that there is a cost in transforming information achieved from the egocentric coordinates into allocentric coordinates [4] which most likely reflect the greater deviations observed in the right and left spaces. It may be argued that the systematic deviations observed in the present study reflect difficulty in forming a purely allocentric representation from haptics, therefore resulting in the perception of a rotated layout. We will further investigate the transformation of egocentric coordinates to allocentric coordinates in experiment 3.

To summarize the results of experiment 1, systematic deviations were also observed in the reproduction of object-to-object relations. The directions of the deviations were different depending on the relative location of the placed object with respect to the other objects fixed in space. These results show that the deviations in the perception of object location are not in a specific direction in relation to the body but when the objects in space show a spatially regular layout, the perception of object locations deviate depending on the relationship among the objects. To further investigate the characteristics of haptic perception of object-to-object relations, we conducted the same experiment on the mid-sagittal plane. Gravity is a major factor which affects arm movement in space. Therefore, if perception of object-to-object relations use a different egocentric reference frame suited for arm movement, then reproduction of object-to-object relations should be affected by gravity.

## Experiment 2

The aim of experiment 2 was to further investigate the formation of spatial representation of object-to-object relations by conducting the layout reproduction task on the mid-sagittal plane. Unlike with the perception of object orientation where the hand is static, orientations concerning object-to-object relations can only be achieved through active arm movement. This in turn means that the main source of information used in the coding of object-to-object orientation is proprioception. Gravity highly affects trajectory planning of the arm, and therefore if different egocentric reference frames to the perception of object orientation are used, then different directional deviations may be observed.

### Method

The experimental procedure was approved by the ethics board of the University of Tsukuba.

#### Participant

Twenty undergraduate and graduate students from University of Tsukuba participated in this experiment (mean age ± SD = 21.89±1.76). Written informed consent was obtained from all individual participants included in the study.

#### Experimental design

A two factor within participant design with reproduction space (shoulder height, above shoulder) and arrangement (oblique, horizontal-vertical) as factors.

#### Stimuli and apparatus

**Fig 7:**
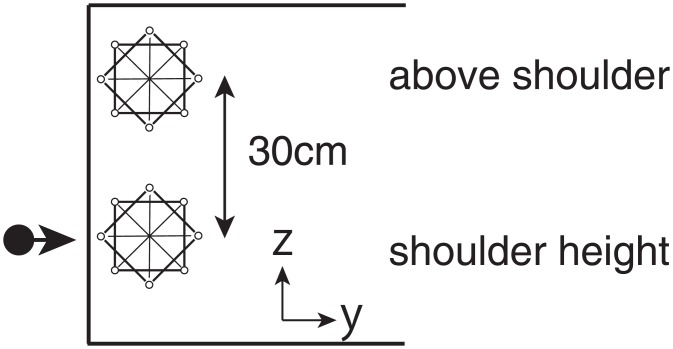
Experimental setup of experiment 2. The black dot depicts the position of the shoulder and the arrow shows the direction the participant is looking. The layouts were presented on a whiteboard positioned on the mid-sagittal plane of the participant. Participants wore eye masks throughout the experiment.

The textures of the stimuli used in experiment 2 were the same as in experiment 1. However, only layouts 1, 3, 5 and 6 of Fig 1 were used in this experiment. The experimental setup differed from experiment 1 in that the task was now conducted on the mid-sagittal plane using a whiteboard as shown in Fig 7. The whiteboard was split into two to resemble the two workspaces: the layouts were presented at shoulder height or above shoulder height.

#### Procedure

Participants sat in front of a table, were informed about the experimental procedure, and gave written informed consent. Afterwards, participants were instructed to move to the whiteboard and were instructed about the experimental procedure in detail. The general task of the participant was the same as in experiment 1. Participants were told that the experiment consisted of a learning phase and a reproduction phase. The general task of the experiment was to learn and reproduce the spatial layout of three magnets placed on the table in front of them while wearing an eye mask.

However, unlike with experiment 1 where the task was conducted on a horizontal plane, experiment 2 was conducted using a whiteboard positioned on the mid-sagittal plane of the participant’s body as shown in Fig 7. All participants used their right hand during the learning and reproduction phases. As only layouts 1,3,5,6 were used, 20 trials were conducted.

### Results

Deviations were recorded in a similar way to experiment 1. Deviations away from the accurate position of the magnet was measured as positive values for the y axis and deviations against the direction of gravity were measured as positive values for the z axis. Angular deviation of the reproduced magnet with respect to the accurate position was measured in the same way as experiment 1. One participant was excluded from the analysis as the data was more than 3SD away from the mean.

#### Magnitude of deviations in reproduction space

A two factor (reproduction space, arrangement) ANOVA was conducted on each axis. There was a significant effect of reproduction space in both axes (y axis: *F*(1,8) = 5.60, *p* < .05; z axis: *F*(1,8) = 18.23, *p* < .01). In both axes, the space above shoulder height had larger absolute deviations than the space at shoulder height. A significant effect of arrangement was also observed for the z axis (*F*(1,8) = 11.61, *p* < .01), with greater absolute deviations in the oblique arrangements as compared to the horizontal-vertical arrangements (Column Exp 2 in Table 1). No interactions between arrangement and reproduction space was observed.

#### Direction of deviations

**Fig 8:**
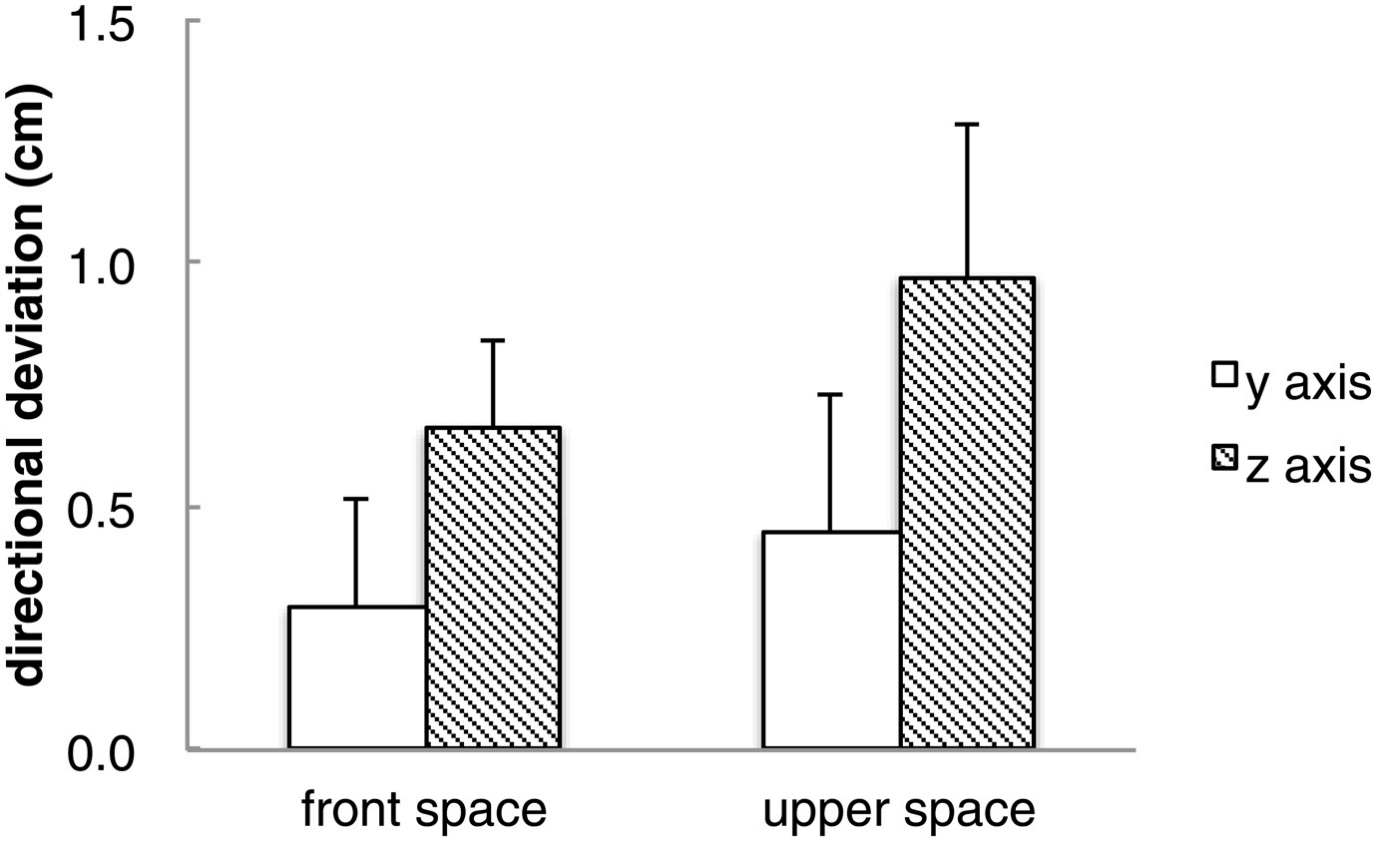
Mean directional deviations for each axis in the front and upper spaces. Error bars show *SE*.

Directional deviation was calculated in the same manner as in experiment 1. However, positive values show deviations in the anti-clockwise direction, as the right hand posture rotates in an anti-clockwise manner when located in space above shoulder height. A one-sample *t*-test was carried out on directional deviation for each axis (y and z) in each reproduction space (shoulder height, above shoulder height). Significant differences to zero were observed for both spaces in the z axis (above shoulder height: *t*(8) = 3.07, *p* < .05; shoulder height: *t*(8) = 3.75, *p* < .01) (Fig 8). Table 3 shows the frequencies of the reproduced magnet in each of the four spaces. It can be observed that the magnets reproduced in space above shoulder height show a trend to be reproduced closer to the body. A chi-squared test of goodness-of-fit was performed to determine whether participants equally reproduced magnets in each area. Reproduction of the magnet was not equally distributed in the four areas for space above shoulder height (χ^2^ (3, *N* = 40) = 16.4, *p* < 0.01) but was equally distributed in the four areas for space at shoulder height. Chi-squared test of goodness-of-fit was performed on each of the four layouts for the space above shoulder height and showed that layouts 3 and 6 were not equally distributed in the four areas (highest frequency for layout 3 was 270~360 degrees and 180~270 degrees for layout 6).

**Table 3:** The frequency of mean trials reproduced in the four areas.

	0~90°	90~180°	180~270°	270~360°
above shoulder	5	3	19	13
shoulder height	14	6	13	7

### Discussion

Deviations were greater when reproduced in space above shoulder height, which was in line with previous research showing that haptic perception of peripersonal space becomes more prone to deviations the further away the objects are from the body. However, the direction of the deviation was different to those observed in experiment 1. Unlike with experiment 1 where higher frequencies of the reproduced magnet were observed in the rotational directional area in the right and left spaces, higher frequencies were observed in areas to the left of the accurate reproduction position in space above shoulder height. Further chi-squared test for goodness-of-fit on the four layouts showed that layouts 3 and 6 showed unequal distributions. Layout 3 showed the highest frequency in area 270~360 degrees and layout 6 showed the highest frequency in area 180~270 degrees. Both of these areas are in the direction of arm movement with respect to the shoulder. Directional deviations only showed significant differences to zero in the z axis direction only, showing that reproduction of object location was more prone to errors in the direction of gravity. Along with the difference in the direction of the deviations, oblique arrangements showed greater deviations than with the horizontal-vertical arrangements, which was analogous to the haptic oblique effect. This difference in arrangement was not observed in experiment 1. The observed difference was most likely because the direction of gravity was in line with one of the sides that constructed the isosceles triangle in the horizontal-vertical arrangements, adding another cue which could be used to judge the to be reproduced position of the object. Gravity acts as a cue in the perception of space using haptics and layouts reproduced further away from the body in the z axis direction were deformed in the direction of arm movement with respect to the shoulder, therefore suggesting that the shoulder might have been used as the egocenter as it is more prone to the effects of gravity.

## Experiment 3

Promotion of accurate spatial perception with non-informative vision is known to reflect a shift in weightings towards the allocentric reference frame, as vision allows participants to relate the proprioceptive positional information to a reference frame defined by Euclidean coordinates (allocentric reference frame). The deviations observed in experiments 1 and 2 reflect the characteristics of the egocentric reference frame used in haptic perception of space. To further establish that this was the case, non-informative vision was introduced as non-informative vision is known to promote shift of weighting to the allocentric reference frame.

### Method

The experimental procedure was approved by the ethics board of the University of Tsukuba.

#### Participant

Ten undergraduate and graduate students from University of Tsukuba were newly recruited to participate in this experiment (mean age = 22.10±2.96). Ten participants were newly recruited to match the number of participants in the right hand group in experiment 1. All participants were right handed. Written informed consent was obtained from all individual participants included in the study.

#### Experimental design

A two factor mixed design with reproduction space (front space, right space) as within participant factor and visual cue (blindfolded (data from experiment 1), table hidden) as between participant factor. The left hand was not tested in this experiment as the main aim of experiment 3 was to observed the effects of non-informative vision.

#### Stimuli and apparatus

The same experimental setup and stimuli were used as in experiment 1. However, unlike with experiment 1, the table was covered with cloth that was positioned at shoulder height so that the participant was not able to see the stimuli presented on the table and his own arm movement but was able to see the table size.

#### Procedure

The experimental procedure was the same as experiment 1, with two exceptions. The participants did not wear eye masks during the experiment, but instead the table was covered with cloth so that the positions of the magnets and the arm movements of the participant were not seen. Participants were also given schematic diagrams of the layouts before the experiment to help participants get familiar with the layouts they were to learn.

### Results

Deviations were recorded in a similar way to experiment 1. Angular deviation of the reproduced magnet with respect to the accurate position was measured in the same way as experiment 1.

#### Magnitude of deviations in reproduction space

A two factor (visual cue, reproduction space) mixed ANOVA was conducted on the absolute errors. A significant main effect of reproduction space was observed in both axes (x axis: *F*(1,9) = 9.08, *p* < .01; y axis: *F*(1,9) = 6.66, *p* < .05) with greater deviation in the right space than in the front space. A marginal significant interaction was also observed in the x axis (*F*(1,18) = 3.19, *p* < .10) and tests on simple main effects showed a significant effect of reproduction space in the blindfolded group (*F*(1,9) = 11.37, *p* < .01) with greater deviations in the right space than in the front space, and a marginal significant effect of visual cue in the right space (*F*(1,18) = 3.69, *p* < .10) with greater deviations in the blindfolded group as compared to the table hidden group (Column Exp 3 of Table 1).

#### Effects of vision on the direction of deviations

**Fig 9:**
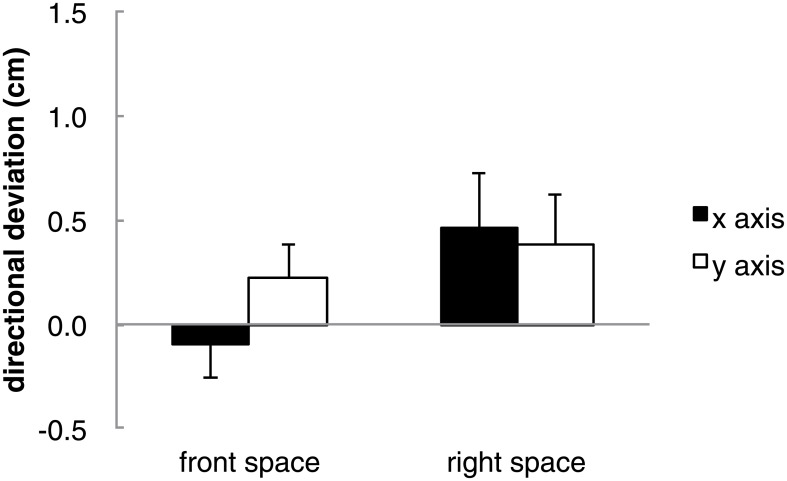
Mean directional deviations for each axis in each space for the table hidden group. Error bars show *SE*.

Directional deviations were calculated in the same way as experiment 1. A one-sample *t*-test was carried out on directional deviation for each axis (x and y) in each reproduction space (front space, right space). No significant difference from zero was observed for either of the axes in either of the reproduction spaces (Fig 9). Table 4 shows the frequencies of the reproduced magnet in each of the four spaces. It can be observed that the magnets reproduced in space above shoulder height show a trend to be reproduced closer to the body. A chi-squared test of goodness-of-fit was performed to determine whether participants equally reproduced magnets in each area. Chi-squared test of goodness showed that participants equally reproduced magnets in each area for the front and right spaces with the help of non-informative vision.

**Table 4:** The frequency of mean trials reproduced in the four areas.

	0~90°	90~180°	180~270°	270~360°
reproduction space (right)	20	22	22	16
reproduction space (front)	24	17	20	19

### Discussion

Participants who only used haptics as the source for perceiving object locations showed greater deviations in the right space. This result shows that non-informative vision promoted accurate reproduction of object locations. This point was further supported by the fact that reproduced object locations of participants who were able to see the experimental setup did not differ from zero in both the front and right spaces. Along with this, angular deviation also did not show any rotational deviations. Participants in experiment 3 were not able to see their hand directly, nevertheless performance in reproducing object locations increased. Spatial information concerning the objects location were not achieved through vision, yet being able to relate proprioception to visual imagery promoted performance. Previous research have shown that the conscious experience of reachable space shows a 10% deviation away from the actual reachable space [36] and can be manipulated by shifted visual feedback [37]. This result confirms that haptic perception of space also uses a weighted average of the egocentric and allocentric reference frame.

## General Discussion

The locational information of the hand and the perceived object is important information for the execution of daily actions such as grasping and reaching. Such actions are executed using spatial representations of the surrounding which are formed from both vision and haptics. A large body of research has shown that haptic spatial perception of peripersonal space is non-veridical. The non-veridical characteristics of haptic peripersonal spatial representations are caused from the limitations of the haptic domain, such as limitations in number of objects that can be touched at a single time and limitations in locations of space that can be perceived due to the posture of the arm. The current study extended on the studies of haptic peripersonal spatial representations by considering perception of object-to-object relations. Investigations into the characteristics of representations of object-to-object relations are important as this is the representation used to carry out reaching movements from one object to another, such as reaching to the navigation panel from the steering wheel while driving. Previous research focused on perception of object orientation and not orientation between objects. Also, previous research concerning perception of object-to-object relations focused mainly on recognition of object locations and not the reproduction of object locations. The present study thus focused on the perception and reproduction of object-to-object relations in order to further understand the characteristics of haptic spatial representations.

Our study showed deformations in the perception of object-to-object relations. Deviations when perceiving object location was not in a specific direction in relation to the body but when the objects in space show a spatially regular layout, the perception of object locations deviate depending on the relationship among the objects. Previous research concerning the perception of location have mainly accounted the perception of an objects position with respect to the body, which is also an object-to-object relation. However, since the body does not move in space, the observed deviations were always in the same direction with respect to the body, away from the body. However, our results suggest that there is no specific direction of deviation in the perception of object location using haptics but that the perceived position of an object changes depending on the relation of the object in respect to other objects in space. This was not the case when object locations were perceived on the mid-sagittal plane as deviations became greater in the direction of gravity. In both experiments 1 and 2, arm movement was a crucial source of information coding the relation of the objects. In experiment 1 the hand could be rested on the table therefore canceling out the effects of gravity when coding the arm position. However, with experiment 2 the hand could not be rested and therefore effects of gravity were greater. This difference was most likely the reason for the different trends in distribution of the reproduced positions between the two experiments, suggesting that a different spatial representations are formed in the perception of object relations on the horizontal plane and on the mid-sagittal plane. It is highly likely that the shoulder was used as the egocenter in perceiving object locations in our experiment, however further experiments with larger number of trials must be conducted so that the distribution of the reproduced magnet position can be analyzed to further confirm this point.

Non-informative visual information increased accuracy in the haptic spatial layout reproduction task like with the perception of parallelity. The effects of non-informative vision showed that interaction with vision helps forming accurate representations of space (Euclidean representation). Dassonville [38] has also shown that delays caused by processing of locational information achieved from proprioception result in a shift of perceived hand position depending on the direction of arm movement and these shifts (or errors) of hand location are corrected with glimpses of the hand between trials or by visual feedback [32,39]. Gandhi, Ganesh, and Sinha [40] have also shown that sight onset late in childhood by means of surgery improve spatial imagery tasks. Even though visual cues in our study were non-informative, visual cues of the surrounding space allowed for locational information achieved from proprioception to be mapped in Euclidean coordinates (allocentric reference frame), promoting shifts in weighting to the allocentric reference frame.

Humans highly rely on vision to achieve spatial information needed to conduct the many actions of daily life. However, vision is not perfect, as there are limitations as to where we may look. There are situations where we are faced with the task of conducting an action without the guide of vision. The main finding of the current study was that perception of object locations in space may differ depending on the relative location of the object in relation to another object. In order for accurate arm movements from one object to another to be carried out in success, further understanding of the formation of spatial representations through haptics, for example factors which define how object positions are perceived as parts of an alignment are needed.
